# Burkitt Lymphoma: beyond discoveries

**DOI:** 10.1186/1750-9378-8-35

**Published:** 2013-09-30

**Authors:** Sam M Mbulaiteye

**Affiliations:** 1 Division of Cancer Epidemiology and Genetics, National Cancer Institute, National Institutes of Health, DHHS, Infections and Immunoepidemiology Branch, 9609 Medical Center Dr, Rm. 6E118 MSC 9704, Bethesda, MD 20892-9704, USA

## Abstract

First described in 1958 in Uganda, Burkitt lymphoma (BL) attracted interest worldwide following reports of its uneven geographic distribution and rapidly fatal clinical course. Both suggested infectious etiology and curability. Seminal discoveries followed in quick succession. Viral etiology – due to Epstein-Barr virus (EBV) – was confirmed. Chromosomal translocations, involving cellular *MYC*, a protooncogene, were discovered, shown to be a hallmark of BL, and central to the genetic basis of cancer. Cure of BL using combination chemotherapy was demonstrated. Unfortunately, civil disturbance in Africa disrupted BL research and blunted its impact on education and oncology care in Africa. Important questions went unanswered. The risk of BL due to malaria or EBV was not quantified. Efforts to answer whether BL could be prevented – by preventing malaria or early EBV infection – were abandoned. The mechanism of malaria in BL is unknown. In Africa, BL remains mostly fatal and diagnosis is still made clinically. Unprecedented advances in molecular, genomics and proteomic technologies, promising to unlock mysteries of cancers, have re-awakened interest in BL. With return of stability to Africa, the unanswered questions about BL are re-attracting global interest. This interest now includes exploiting the knowledge gained about genetics, proteomics, and bioinformatics to enable the development of targeted less toxic treatment for BL; and simpler methods to diagnose BL with high accuracy and sensitivity. The articles in the *Burkitt Lymphoma (BL): Beyond Discoveries* in *Infectious Agents and Cancer* highlight BL as priority. Authors explore etiology, pathology, pathogenesis of BL, and whether knowledge gained in the studies of BL can catalyze sustainable cancer services in one of the world’s poorest served regions.

## Burkitt lymphoma: beyond discoveries

Infectious Agents and Cancer is pleased to present a thematic series entitled *Burkitt Lymphoma (BL): Beyond Discoveries*. BL is an aggressive non-Hodgkin lymphoma (NHL) [1] that was first described in African children by Dennis Burkitt [2]. Within less than 10 years, Burkitt’s report of an obscure tumor in Africa had a dramatic impact on epidemiology, virology, immunology and oncology, spawning over 10,000 publications within a few decades of it’s description in 1958 [3]. Originally considered peculiar to Africa, characterization of histochemical and cytological properties of BL [1,4,5] led to recognition of cases worldwide and realization that the discovery had a universal relevance [6]. Notable geographic differences in BL incidence were apparent and suggested etiology by a vectored virus [7]. They also suggested an intuitive classification of the types of BL as “endemic BL “when occurring at a high incidence and “sporadic BL” when occurring at low incidence [7].

**Figure 1:**
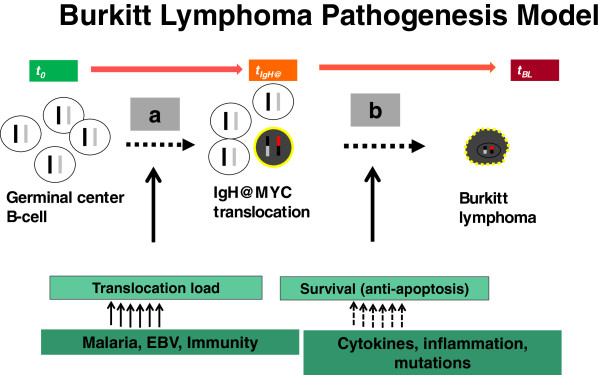
**A cartoon showing a simple pathogenesis model of Burkitt lymphoma (BL) showing progression from a naïve B cell through a necessary pre-malignant stage involving chromosomal translocation of MYC on chromosome 8 into the vicinity of promoter elements of immunoglobulin genes on chromosome 14, 2, or 22 and progression of translocation-positive B cells to a clone of malignant BL.** The first stage is indicated by letter **a** and the second stage by letter **b**. The transit times for these stages are unknown, but several assumptions are possible. First, exogenous exposures linked to high risk of BL, such as infection with malaria, Epstein-Barr virus, and human immunodeficiency virus (HIV) – in the West – may act by increasing the absolute number (load) of translocation-positive B cells, which would increase the number of initiated cells that can progress to BL and, hence, population incidence of BL. Second, the rate-limiting step of BL is the apoptosis feedback loop in translocation-positive B cells. Thus, exposures that increase the survival, i.e., circumvent apoptosis feedback loops in translocation-positive B cells until the abnormal cells develop capacity for self-perpetuation will increase the individual risk of BL.

Study of BL led to a quick succession of seminal discoveries. Epstein-Barr virus (EBV) was discovered in 1964 in tumor cells cultured from an African case examined by electron microscopy [8]. Dramatic response to chemotherapy and cure of BL was reported and replicated in trial and error efforts [9,10]. Chromosomal translocations involving light and heavy chain immunoglobulin genes and *MYC* were discovered in BL tumors [11,12], unlocking new ways to study tumor biology (Figure 1). The study of mouse tumors with analogous translocations - plasmacytomas -became an important resource to developing monoclonal antibodies. In 1969, after epidemiological studies confirmed that EBV, the virus found in BL tumors, was not transmitted by a vector [13], Dennis Burkitt proposed recurrent infection with *Plasmodium falciparum* (*Pf)* as co-factor in BL etiology [14]. Today, BL is considered a model disease to understand the poly-microbial and the genetic basis of cancer [15]. Specifically, a pathogenesis model can be constructed (Figure 1), where the nodes of risk comprise of exposures to infections that may increase the risk of developing chromosomal translocation and exposures that confer longevity to translocation-positive B cells by circumventing apoptosis feedback loops induced by overexpression of c-*MYC*.

Many questions about BL remain unanswered. For example, the definition of BL and its subtypes remains a matter of debate [16]. Dennis Burkitt described a purely clinical entity in Africa [2]. In 1969, a consensus conference organized by the World Health Organization (WHO) in Washington D.C. reviewed tumor tissues from patients with clinical BL and reached a consensus definition that BL was a distinct patho-biological entity characterized by specific histological features or properties [17]. This pathologic definition has changed over the years as pathology techniques have evolved. Refinements in histochemical stains, morphological, cytogenetic, immunophenotypic and, molecular techniques have led to description of classical cases, variously referred to typical or classical BL, and variant cases referred to as atypical BL or BL-like. The pathology properties noted above are unrelated to the intuitive clinico-epidemiological classification of BL as endemic, sporadic, and immunodeficiency associated BL.

The infectious etiology of BL was recognized immediately. Despite this, until the advent of the HIV epidemic and application of organ transplant technologies, which necessitated immunosuppressive therapy to prevent organ rejection, no clear evidence linking immunosuppression in individuals to the risk of BL was adduced [18]. Other provocative questions went unanswered. These include why jaw tumors occur significantly more commonly in males than females, particularly in prepubescent children [19]. Because BL is endemic in Africa, it has always been assumed that basal risk for BL is higher in Blacks even in areas where BL is sporadic. However, data from the U.S., where many Blacks live and BL is sporadic suggest that the risk of BL may be lower in Blacks than Whites [20,21]. Why? Blacks in the U.S. are more likely to be of a lower socioeconomic status and therefore more likely to be exposed to EBV at a younger age [22]. Assuming EBV conveys an independent risk of BL risk, one would predict that the risk of BL would be higher in Blacks than in Whites. Yet the opposite has been repeatedly observed, even in the setting of immunosuppression [23,24]. If EBV is relevant, then is early exposure to EBV in the absence of malaria protective for BL? If EBV is irrelevant, then do Blacks have a lower basal genetic risk of BL? Or are there other local exposures in Africa that are the culprit? Likewise, the higher risk of BL in young males compared to females, observed in endemic, sporadic, and immunodeficiency BL is an unsolved puzzle. Answers to these questions could provide new clues about lymphoma biology. The endemicity of BL maps quite well with holo-endemic malaria, in and outside of Africa (example, Papua New Guinea). But if early acquisition of EBV and malaria together increase risk for BL, why is BL not endemic in other malarial regions, such as in India and Burma? Are there differences in risk posed by *P. falciparum* and *P. vivax*? Are differences in clinical presentations and EBV association of endemic and sporadic BL indicative of truly different clinical entities? Are EBV positive jaw tumors seen in 'non endemic” settings like Turkey [25] similar to disease in Africa?

**Figure 2:**
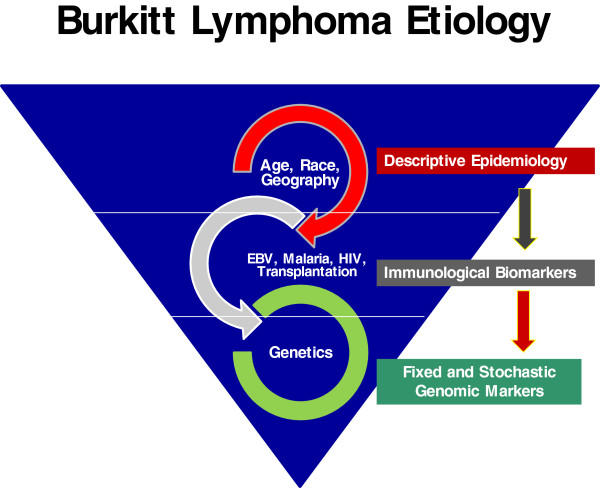
**A schema showing studies of multi-factorial etiology of Burkitt lymphoma (BL).** Descriptive epidemiological studies have demonstrated sex, age, and geography as risk factors. Immunological and biomarker studies have demonstrated increased risk related to infections that disrupt immunity, such as malaria, human immunodeficiency virus, and use of solid organ transplantation as risk factors. The genetic basis of BL remains largely explored and represents the next frontier in BL research.

The treatment approaches of BL in the West reflect lessons learnt from trial and error efforts treating endemic BL [26]. Today, treatment of BL is highly effective in the West and response and cure rates to present day regimens are >90% [27]. In Africa, long-term cure rates have remained low and have not moved beyond rates of 25-30% described in early days [28]. While the reasons for poor outcomes are due to limited resources and capacity, it is also possible that many cases are either not spotted or are diagnosed late and when diagnosed and treated, they abandon treatment [29]. Would efforts to intensify case spotting, early diagnosis and referral to treatment centers significantly affect mortality in Africa [30]? Moreover, current BL treatment is associated with life-threatening side effects, which complicate its use in Africa where intensive-care services are basic [31]. Would discovery of newer drugs, but with fewer side effects increase uptake and bring lifesaving benefits to African children [32]. How could our understanding of the genetic and the poly-microbial basis of BL be harnessed for treatment and prevention? How EBV and malaria cause BL remains poorly understood [3,33]. Clarifying the biological pathways exploited by these infections to influence risk for BL (Figure 1) could unlock ways to diagnose [34], treat and or prevent BL. The outstanding of bottlenecks to BL research illustrated by the cartoon include lack of methods to measure the prevalence and load of c-*MYC* translocation-positive B cells reliably and reproducibly in asymptomatic individuals [11,35]. Development of sensitive tests that can be used in epidemiologic studies could clarify the role of malaria, EBV, and other co-factors. Secondly, such tests could open new avenues for investigation, including cohort studies of children with a high load of c-*MYC* translocation-positive B cells. While the brief review indicates some understanding of epidemiological and immunological risk factors of BL (Figure 2), our understanding of the genetic basis of BL still remains rudimentary. The interest in BL that is re-emerging is timely because the unprecedented advances in molecular, genomics and proteomic technologies could unlock the mysteries of BL and deliver on its promise as the Rosetta Stone of Cancer [36].

Through the articles in *Burkitt Lymphoma (BL): Beyond Discoveries, Infectious Agents and Cancer* aims to highlight BL as a priority, which allows unique opportunities for scientists, policy makers, program managers in countries where BL is endemic to collaborate. Their challenge will be to answer to what extent the discoveries made about BL will impact the life of a child with or at risk of BL in Africa. Can knowledge gained in the studies of BL, conducted both in African countries solely outside Africa, be translated into affordable implementable programs that introduce or catalyze sustainable cancer services in one of the world’s poorest served regions [37]. Some of these questions are not be new, but there are new toolboxes in the present genomic era to answer new and old questions. These issues and others, not explicitly touched, will be discussed in the various papers collected in the thematic issue introduced here.
